# Functional capacity in smoking patients after coronary artery bypass grafting surgery: a quasi-experimental study

**DOI:** 10.25122/jml-2023-0282

**Published:** 2023-12

**Authors:** Mohammed Essa Alsubaiei, Wadha Althukair, Hind Almutairi

**Affiliations:** 1 Department of Physical Therapy, Faculty of Applied Medical Sciences, Imam Abdulrahman bin Faisal University, Dammam, Kingdom of Saudi Arabia; 2 Department of Physical Therapy, Saud Al-Babtain Cardiac Center, Dammam, Kingdom of Saudi Arabia; 3 Department of Quality Improvement and Patient Safety, Dhahran General Hospital for Long Term Care, Dhahran, Kingdom of Saudi Arabia

**Keywords:** functional capacity, coronary artery bypass grafting surgery, smoking

## Abstract

Coronary artery bypass grafting surgery (CABG) is an important approach to treating coronary heart disease. However, patients undergoing open heart surgery are at risk of postoperative complications. Cigarette smoking is one of the preoperative risk factors that may increase postoperative complications. Studies show that early mobilization intervention may reduce these complications and improve functional capacity, but the impact of smoking on early outcomes after CABG has been controversial for the past two decades. This quasi-experimental study explored the effects of early mobilization on functional capacity among patients with different smoking histories undergoing CABG. The study involved 51 participants who underwent CABG surgery, divided into three groups: current smokers, former smokers, and non-smokers (n=17 each). A day before surgery, all groups underwent a six-minute walking test (6MWT). Every participant received the same intervention after surgery, including deep breathing exercises, an upper limb range of motion assessment, an incentive spirometer, and walking with and without assistance. Five days postoperatively, all outcomes - including the 6MWT, length of stay (LOS) in the ICU, and postoperative pulmonary complications - were assessed, and the 6MWT was repeated. There was a reduced functional capacity after CABG in ex-smokers (215.8±102 m) and current smokers (272.7±97m) compared to non-smokers (298.5±97.1m) in terms of 6MWT (p<0.05). Current smokers were more likely to have atelectasis after CABG than ex-smokers (76.5% vs. 52.9%), with non-smokers being the least likely to have atelectasis among the three groups (29.4%, p<0.05). Additionally, current smokers required longer ventilator support post-CABG (11.9±7.3 hours) compared to ex-smokers (8.3±4.3 hours) and non-smokers (7±2.5 hours, p<0.01). Smoking status significantly impacts functional capacity reduction after CABG, with current smokers being more susceptible to prolonged ventilator use and atelectasis.

## INTRODUCTION

Coronary artery disease (CAD) accounts for more than 375,47 deaths in the United States (US) alone [1]. In 2004, a community-based study published in Saudi Arabia found that the general prevalence of CAD was 5.5% [2]. Another pilot study found that 32% of the population were diagnosed with ischemic heart disease. The same study also reported the most prevalent risk factors for CAD, which included obesity (49.6%), diabetes (25.1%), hypertension (30.3%), persistent smoking (12.2%), and hyperlipidemia (32.1%) [3]. However, this percentage is quite lower than what the American Heart Association (AHA) reported between 2011 and 2012 when they claimed that 9.4% of US adults had a 10-year predicted risk of cardiovascular disease (CVD) [4]. Furthermore, CAD is considered the most common underlying pathophysiology for heart failure (HF) within industrialized nations [5].

Coronary artery bypass grafting surgery (CABG) is considered an important approach for treating coronary heart disease. After open heart surgery, patients are at risk of postoperative complications, such as death and wound complications [6-8]. For the past decade, the focus on preventing these complications by improving surgical techniques has remarkably decreased mortality rates [9], although they remain elevated compared to other types of surgery [10]. Numerous preoperative risk factors may increase the chances of postoperative complications, including gender, age, diabetes, metabolic syndrome, hypertension, renal failure, poor left ventricular ejection fraction (EF), previous CABG, the extent of CAD, a recent myocardial infarction, urgent operation, and smoking [11-19].

Tobacco smoking is considered one of the leading global causes of preventable mortality and morbidity [20]. It is a pre-exposing factor that increases the risk of peripheral vascular disease, CAD, and chronic obstructive pulmonary disease (COPD) [21-23]. In addition, the prevalence of persistent smoking in Saudi Arabia ranges from 2.4% to 52.3%, depending on age and sex, based on an article reviewed and published in 2009 [24]. In 2013, a survey of 10,735 participants revealed that the prevalence of current smokers was 12.2%, and daily shisha smokers accounted for 4.3% of the population. In the same report, daily smokers of both cigarettes and shisha comprised 1.4% of smokers [25]. The percentage is comparable with a report published by AHA in 2015 that found rates of adult tobacco consumption estimated at 15.2% [26]. However, smoking cessation is well-associated with significantly improved health outcomes [27]. The impact of smoking on early outcomes after CABG has remained controversial over the past two decades; some articles have recommended that CABG be avoided amongst persisting smokers due to an increased risk of post-surgical mortality and complications, as well as vein graft early failure that may lead to early re-operation [28-30]. Nevertheless, there has been recent evidence that smoking may not be positively related to early outcomes after CABG [31-33].

To date, there has been no published article discussing the relationship between smoking and postoperative exercise capacity, although one article discussed the association between smoking and normal exercise capacity [34]. The average 6MWT score in the non-smokers group was higher than the active smokers (never-smokers: 554.5m (516.5–606.0m) vs. active smokers: 515.5m (477.0–570.0m), p=0.004. Despite the decrease in functional capacity in smokers compared to non-smokers [34], there is no verified link between smoking and early mobilization after CABG. It is expected that smoking may decrease the functional capacity of patients with CAD.

The effect of smoking on postoperative pulmonary complications (PPCs) has been addressed in a few articles. One serious set of complications patients face is pulmonary complications resulting from the disruption of normal ventilation [35]. Pulmonary complications vary and may include pneumonia or atelectasis [36]. The effects and outcomes of PPCs also differ, from increased financial costs and prolonged stay in hospital to increased major morbidity and mortality [37-40]. An Australian study investigating pneumonia and prolonged ventilation (for more than 24 hours) found that smokers were two times more likely to be diagnosed with pneumonia after CABG compared to quitters and non-smokers (odds ratio (OR)=2.05, 95% confidence interval (CI), p<.001) [41]. On the other hand, prolonged ventilation showed a weak association with smoking (OR=1.19, 95% CI, p=.044). Research has shown that early mobilization interventions, involving the prompt mobilization of patients and encouraging them to move as soon as possible after surgery, may reduce postoperative complications and improve functional capacity, which refers to the individual's ability to perform activities of daily living such as walking [7, 42-44]. A study compared an early mobilization intervention group with a control group [45]. The intervention group showed a lower rate of pleural, atelectasis, and length of in-hospital stay.

Furthermore, patients in the control group achieved less distance in the 6MWT at the time of discharge (from 343±64 to 272±52 m) when compared to the intervention group (from 316±58 to 299±72 m). Santos *et al*. stated, in their systematic review, that any early mobilization program after surgery would have a positive effect on postoperative outcomes [46]. The literature shows the presence of selection bias, as the studies include all ages, different body mass indexes (BMIs), HF, and COPD, which are independent risk factors for postoperative complications. In our study, we controlled for these factors by establishing well-defined inclusion-exclusion criteria, focusing on smoking status as the main factor. Another indication of bias is the absence of similar research in the Middle East, where lifestyle, culture, and the prevalence of different risk factors vary significantly and where other types of smoking are popular. Therefore, this study aimed to explore the effects of early mobilization on functional capacity in patients who smoke and have undergone coronary artery bypass grafting (CABG), comparing outcomes among current smokers, former smokers, and never-smokers.

## MATERIAL AND METHODS

### Study design and setting

This quasi-experimental study was conducted using data from Saud Al-Babtain Cardiac Center, Dammam, Saudi Arabia.

### Sample size calculation

Based on a pilot study (n=21) showing a significant reduction in the 6-minute walk test (6MWT) after CABG surgery across different smoking groups, we calculated a minimal sample size of 51 patients (17 per group). We assumed a Type I error of 5% (α=0.05), an effect size F of 0.58, and a power of 95% (1- β), as recommended by previous guidelines [47].

### Inclusion and exclusion criteria

In this study, male patients were exclusively included due to the low eligibility rate of female patients at Saud Al-Babtain Cardiac Center. The age range was limited to 35-65 years to minimize age-related preoperative risk factors [13, 14]. All participants were scheduled for isolated, elective coronary artery bypass grafting (CABG) surgery. We also restricted the BMI criteria between 20 to less than 30 kg/m^2^ to reduce the impact of obesity as a preoperative risk factor [48]. Furthermore, eligibility criteria required hemodynamic stability, as confirmed by a physician based on the patient's blood pressure, heart rate, and peripheral perfusion. Patients were excluded from the study if they had COPD and pulmonary hypertension, preoperative HF or EF less than 30%, any neurological or neuromuscular disorder, the need for an intra-aortic balloon pump, undergoing additional procedures or operations during Intensive Care Unit (ICU) stay and admission for emergency surgery.

### Outcome measures

#### Six-minute walk test

The 6MWT was conducted twice for each participant: the day before surgery and the fifth postoperative day before hospital discharge. This approach allowed for comparison both within the same group and between different groups. The test was performed in a 100-foot (30.48-meter) corridor following the American Thoracic Society (ATS) guidelines [49]. The researcher (WA) instructed the subjects to walk as fast as possible and recorded oxygen saturation, heart rate, blood pressure, and the Borg dyspnea scale based on the ATS guidelines criteria before and after the test. Walking distance was recorded in meters [49, 50]. The test is considered valid and reliable to evaluate exercise capacity in subjects after cardiac surgery [51].

#### Postoperative pulmonary complications

Two PPCs were assessed: atelectasis and prolonged postsurgical mechanical ventilation because these are the most common PPCs reported in previous studies [52, 53]. An independent ICU consultant was responsible for the atelectasis diagnosis, as defined by radiological and clinical criteria, including symptoms and physical examination [54]. This same consultant also made decisions regarding postoperative mechanical ventilation, measured in hours.

#### Postoperative length of stay in ICU

The length of stay (LOS) in the ICU was calculated from the time of patient admission immediately after surgery until discharge by the ICU team. Discharge criteria included improvement and stabilization of the patient's physiological status, with no further need for critical monitoring or care, no plans for additional active interventions, stable hemodynamic parameters, and satisfactory condition of wound and catheter sites.

### Patient recruitment and smoking status classification

All patients were recruited by direct referral from the cardiac surgery department. After each patient was scheduled for surgery, his file was examined for eligibility. The first 17 in each group that met the criteria were recruited. The researcher, WA, contacted the patient and asked him to sign a consent form after providing a participant information sheet and ensure the participant understood all information. After that, each patient was categorized as a current smoker, ex-smoker, or non-smoker [20]. A current smoker was defined as an individual who smoked water pipe, cigarette, or e-cigarette within the past month. An ex-smoker was defined as someone who abstained from any form of smoking for at least a month before surgery but had a history of smoking. A never-smoker was defined as someone with no history of smoking in any form and whose value for the pack-per-year variable was equal to 0. In addition, participants were asked three questions regarding their smoking history:

1. How many cigarettes per day have you smoked?
2. How many years have you used tobacco?
3. Do you smoke cigarettes, e-cigarettes, water pipes, or a combination?

### Preoperative and postoperative procedures

The intervention started one day before the surgery, based on a study by Hirschhorn *et al*. [53]. Independent physiotherapists, unaware of the participants’ smoking status, administered the intervention. After categorizing patients into current smokers, former smokers, or non-smokers, each participant underwent the 6MWT under the supervision of the researcher. This was done to establish baseline functional capacity the day before surgery [55]. The test occurred between 1 and 4 pm for all patients, ensuring at least one hour of rest after a meal. They were also briefed about the surgery and physical therapy protocols. After surgery, patients were transferred to the surgical ICU. Mechanical ventilation time post-surgery was recorded in hours. Once patients were extubated from mechanical ventilation, all groups underwent the physical therapy intervention conducted by an independent physiotherapist, which included walking, strength, endurance, and breathing exercises. An education session was provided as well.

### Atelectasis, mechanical ventilation, and ICU stay

The presence of atelectasis was determined by a blinded ICU consultant on the first day after extubation. The duration of postsurgical mechanical ventilation was recorded in hours by respiratory therapists as part of routine care immediately after extubation. The LOS in the ICU was determined from the time noted by the ICU nurse upon patient admission post-surgery until the transfer note written by the nurse in the surgical step-down unit. Both the mechanical ventilation time and ICU LOS were meticulously calculated and extracted from each patient's file.

### Six-Minute Walk Test

The 6MWT was integral for comparing preoperative and postoperative functional capacity within and between the three patient groups (current smokers, former smokers, and non-smokers). The researcher, WA, conducted both data collection and the 6MWT. Each measurement of the 6MWT was performed at the same time of day, at least one hour after the most recent meal. Patients were instructed to wear comfortable footwear and perform the test without a face mask. Finally, the physical therapy team agreed not to give an afternoon session to the participants. Patients were also instructed to avoid any strenuous physical activity in the hour leading up to the test.

### Safety measures for high-risk cases

Illiterate subjects were accompanied by a family member or another witness. Also, translators (usually from the nursing team) were present for participants who did not speak Arabic or English. No human assistance/support was provided during the 6MWT tests. The primary physiotherapist and the investigator were present to supervise and ensure the corridor was empty and clean, with two separate chairs in place. Moreover, subjects with disturbed balance had an assistant on either side while walking. They were also followed by a wheelchair if needed.

### Statistical analysis

Statistical analysis was performed using SPSS (IBM Corp. Released 2015. IBM SPSS Statistics for Windows, Version 23.0. Armonk, NY: IBM Corp). The normal distribution of the data was tested using the Shapiro-Wilk test. Continuous variables were described as mean ± standard deviation and were compared between the three groups using the one-way ANOVA and Kruskal-Wallis tests based on the satisfaction of the normal assumption. On the other hand, categorical variables were described as numbers and percentages and were compared between the three groups using the chi-square (χ2) test. In addition, the paired samples statistic method was used to identify the differences between the three groups in 6MWT before and after CABG. The effect size of smoking on each outcome variable was quantified by η^2^ (Eta squared). The p-values were considered statistically significant when less than 0.05.

## RESULTS

**Figure 1:**
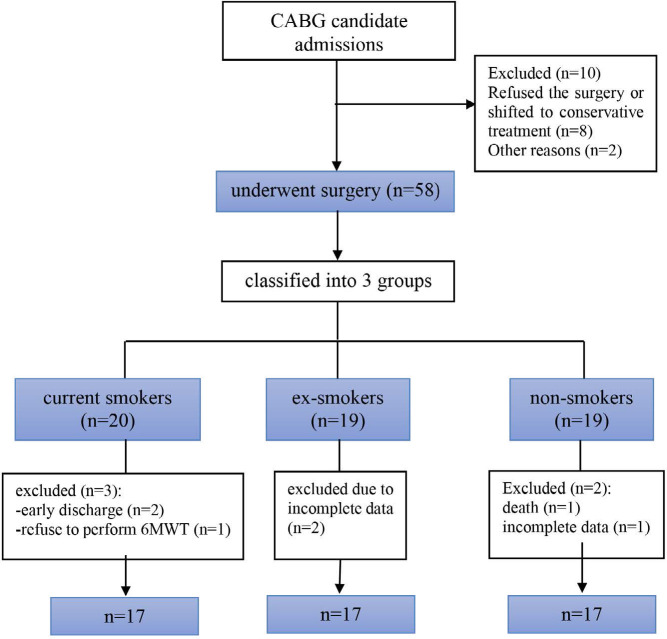
Flowchart illustrating the progression of patients throughout the study period

The data were collected from June 2020 to February 2021. Initially, 68 patients signed the consent forms to be included in the study. After that, 10 were excluded because they refused the surgery or shifted to medical treatments (n=8), and two for other reasons (Figure 1). A flowchart indicating the progression of patients through the study period is shown in Figure 1.

The final study sample comprised 51 male patients. The age range of these participants was 39 to 65 years, with an average age of 54.2±7.1 years. Preoperative factors and patient characteristics that showed significant and insignificant differences among the three groups are summarized in Table 1. Age and type of diabetes mellitus were the only factors that showed significant differences among the three groups.

**Table 1:** Sociodemographic and clinical variables at admission among the three groups that underwent CABG (n=51)

Variable	Non-smokers (n=17)	Ex-smokers (n=17)	Current smokers (n=17)	p-value
**Age (mean± SD)**	54.6±5.9	57.1±6.5	50.8±7.7	0.032*
**Body mass index (BMI) (mean±SD)**	28.3±1.3	27.9±2.2	25.9±3.2	0.063
**Ejection fraction (mean± SD)**	48.7±10.8	47.8±10.3	48±11.7	0.967
**Type of procedure**				
off-pump	5 (29.4%)	2 (11.8%)	5 (29.4%)	0.375
on-pump	12 (70.6%)	15 (88.2%)	12 (70.6%)	
**Smoking type**				
cigarette	-	16 (94.1%)	13 (76.5%)	0.315
electronic cigarette	-	0 (0%)	0 (0%)	
water pipe (sheesha)	-	0 (0%)	3 (17.6%)	
mixed type	-	1 (5.9%)	1 (5.9%)	
**Smoking (packets per year)**	-	37.8±24.0	52.8±30.1	0.118
**Diabetes mellitus**				
None	3 (17.6%)	0 (0%)	7 (41.2%)	0.003**
Type 1	0 (0%)	6 (35.3%)	3 (17.6%)	
Type 2	14 (82.4%)	11 (64.7%)	7 (41.2%)	
**Preoperative hypertension**				
Absent	6 (35.3%)	4 (23.5%)	6 (35.3%)	0.695
Present	11 (64.7%)	13 (76.5%)	11 (64.7%)	
**Number of grafts (mean± SD)**	3.2±0.9	3.3±0.8	3.18±0.8	0.490
**Hypercholesterolemia**				
No	4 (23.5%)	4 (23.5%)	9 (52.9%)	0.110
Yes	13 (76.5%)	13 (76.5%)	8 (47.1%)	
**Renal failure**				
No	16 (94.1%)	17 (100%)	17 (100%)	0.361
Yes	1 (5.9%)	0 (0%)	0 (0%)	
**Recent MI**				
No	16 (94.1%)	17 (100%)	16 (94.1%)	0.594
Yes	1 (5.9%)	0 (0%)	1 (5.9%)	
**Peripheral vascular disease**				
No	17 (100%)	16 (94.1%)	17 (100%)	0.361
Yes	0 (0%)	1 (5.9%)	0 (0%)	
**History of congestive HF**				
No	17 (100%)	17 (100%)	17 (100%)	-
Yes	0 (0%)	0 (0%)	0 (0%)	
**Unstable angina**				
No	17 (100%)	16 (94.1%)	15 (88.2%)	0.346
Yes	0 (0%)	1 (5.9%)	2 (11.8%)	
**LMS>50%**				
No	16 (94.1%)	17 (100%)	13 (76.5%)	0.056
Yes	1 (5.9%)	0 (0%)	4 (23.5%)	
**Lung disease**				
No	16 (94.1%)	17 (100%)	16 (94.1%)	0.594
Yes	1 (5.9%)	0 (0%)	1 (5.9%)	
**Preoperative 6MWT (mean±SD)**	406.7±90.2	349.9±99.9	389.3±70.8	0.166

** Statistically significant (p<0.01); *Statistically significant (p<0.05) MI: myocardial infarction; HF: heart failure; LMS: left main coronary artery stenosis; 6MWT: six-minute walking test (meters)

Current smokers were more likely to be younger than ex-smokers, with non-smokers showing an intermediate pattern between the two groups (Table 1). Non-smokers were more likely to have type 2 diabetes than ex-smokers (82.4% versus 64.7%), with current smokers being the least among the three groups (41.2%, p<0.01).

Significant differences were observed among the three groups in terms of postoperative exercise capacity (p<0.05), atelectasis incidence (p<0.05), and the need for mechanical ventilation (p<0.01) following CABG surgery, as detailed in Table 2. However, there were no significant differences in the LOS in the ICU among the groups (p>0.05). In what concerns postoperative exercise capacity, as measured by the 6MWT, non-smokers demonstrated a higher capacity (298.5±97.1 m) compared to current smokers (272.7±97.1 m). Ex-smokers exhibited the lowest exercise capacity among the three groups (215.8±102.8 m, p<0.05).

**Table 2:** Outcomes after CABG surgery among the three groups (n = 51)

Outcome	Non-smokers (n=17)	Ex-smokers (n=17)	Current smokers (n=17)	p-value	Effect size η^2^
**Postoperative 6MWT (mean ± SD)**	298.5±97.1	215.8±102.8	272.7±97.1	0.045*	0.115
**Atelectasis**					
No	12 (70.6%)	8 (47.1%)	4 (23.5%)	0.023*	0.148
Yes	5 (29.4%)	9 (52.9%)	13 (76.5%)		
**Mechanical vent (hours)**					
Mean ± SD	7±2.5	8.3±4.3	11.9±7.3	0.009**	0.15
**ICU stay (hours)**					
Mean ± SD	71.45±24.3	69.6±26	85.6±37.5	0.355	-

** Statistically significant (p<0.01); *Statistically significant (p<0.05) MI: myocardial infarction; HF: heart failure; LMS: left main coronary artery stenosis; 6MWT: six-minute walking test (meters); ICU: intensive care unit

As shown in Table 3, there were significant differences between preoperative and postoperative exercise capacity for each group (p<0.001), as determined by the 6MWT results. All t statistic values were negative, meaning that each group had a significant reduction in their 6MWT distance after CABG. Moreover, current smokers were more likely to have atelectasis after CABG than ex-smokers (76.5% vs. 52.9%), with non-smokers being the least likely to have atelectasis among the three groups (29.4%, p<0.05). Moreover, current smokers were more likely to stay on the ventilator after CABG than ex-smokers (11.9±7.3 versus 8.3±4.3), with non-smokers being the least likely to stay on the ventilator after CABG (7±2.5, p<0.01). However, the values of η^2^ (Eta squared) indicate that the size effect of smoking on atelectasis and mechanical ventilation was high (η^2^=0.148; 0.15 respectively) and medium on postoperative exercise capacity (η^2^=0.115) (Tables 2 and 3).

**Table 3:** Differences in preoperative and postoperative 6MWT tests for the three groups (n=51)

Group	Preoperative 6MWT	Postoperative 6MWT	t^a^	p-value
	Mean ± SD	Mean ± SD		
**Non-smokers (n=17)**	406.7±90.2	298.5±97.1	-5.542	<0.001**
**Ex-smokers (n=17)**	349.9±99.9	215.8±102.8	-5.647	<0.001**
**Current smokers (n=17)**	389.3±70.8	272.7±97.1	-7.339	<0.001**

a . Paired samples statistic; **Statistically significant (p<0.01)

## DISCUSSION

The main finding of this study was that smokers had a different functional capacity in terms of 6MWT after CABG compared to non-smokers and ex-smokers. In addition, current smokers were more susceptible to common postoperative pulmonary complications than other groups. On the other hand, the LOS in the ICU was not associated with smoking status, which is consistent with other studies.

In this study, current smokers were more likely to be younger than ex-smokers, with non-smokers showing an intermediate pattern between the two groups. This aligns with findings from several studies indicating a higher prevalence of smoking among younger patients with coronary artery disease under 55 years [56, 57]. Only one cross-sectional study investigated the effect of smoking on the 6MWT [34]. This study found that current smokers had more impaired exercise capacity than non-smokers but better results than COPD patients. However, the inclusion criteria of that study encompassed a wide range of ages, EF, and BMI, potentially introducing selection bias, especially since the COPD group had a higher mean age of 64.5 years (ranging from 58 to 74.5 years). Also, the cohort in the Caram *et al*. study did not undergo any type of surgery [34]. On the other hand, the well-defined inclusion criteria in this study reduced the influence of other independent risk factors, like age, BMI, EF, and COPD. These differences may explain the difference in results.

Few studies have attempted to examine the effects of smoking on post-operative atelectasis as an independent risk factor. One prospective observational study was done on patients after lung resection via thoracotomy [58]. This study showed that smokers were seven times more likely to have PPC, including atelectasis, which might support our results. Another retrospective study that analyzed patients undergoing renal transplantation surgery found a correlation between atelectasis and smoking [41]. In addition, they detected a positive and strong correlation between atelectasis and pack per year, which also aligns with our results.

Another key outcome we examined was the necessity for mechanical ventilation post-CABG. Ji *et al*. found that smokers had a longer duration of mechanical ventilation, with a mean ventilation time of 9.9±2.7 h compared to former smokers (9.5±2.3 h) and non-smokers (9.4±2.4 h) [57]. This observation is in line with our results. Moreover, other studies have identified a link between PPCs and 6MWT performance after elective abdominal or thoracic onco-surgery and pulmonary surgeries [59, 60]. This may help explain why both current and ex-smokers in our study showed poorer outcomes compared to non-smokers.

In this study, we found no association between smoking status and LOS in the ICU, a finding consistent with other research [57, 61]. However, Saxena *et al*. reported a significant difference in ICU LOS post-procedure among current smokers, former smokers, and non-smokers (49.10±125.61 h, 43.35±71.67 h, and 42.42±125.61 h, respectively, p=.003) [41]. Atoui *et al*. identified several pre-operative risk factors linked to prolonged ICU stay following cardiac operations, including an EF below 40%, renal failure, older age, female gender, high Parsonnet scale scores, and emergency surgeries [62]. Contrarily, a study reported that patients under an early extubation protocol within six hours post-surgery experienced longer ICU stays [63], which contradicts Zarrizi *et al*.'s findings that ICU LOS is influenced by the occurrence of atelectasis, the presence of more than two chest tubes, and post-surgical atrial fibrillation [64]. Additionally, another study highlighted pre-operative risk factors such as older age, female gender, higher BMI, and poor mobility [65]. All these variables and risk factors may explain the lack of association between smoking and LOS in the ICU.

The main focus of this study was to limit the reduction of functional capacity after CABG and to compare different smoking groups that underwent the same intervention protocol. Another study had a similar intervention and showed that the 6MWT for the breathing/exercise intervention group (431±98 m) was significantly better than the standard group (377±90 m, p=0.022) [54]. The differences in results between the previous study and our study may be attributed to the inclusion of stair climbing (both ascending and descending) in their protocol for all groups, an element that was not part of our study's protocol due to hospital policy. Another article compared an intervention group with a control group [66]. Patients in the control group achieved less distance in the 6MWT at the time of discharge (from 343±64 to 272±52m) compared to the intervention group (from 316±58 to 299±72m). The differences in these findings compared to our study might be due to their protocol, which involved pre-operative testing for five days and included stair climbing post-surgery. Another study that contradicts our findings involved aerobic exercise with a cycle ergometer for 10-20 minutes daily in addition to walking [66].

This is the first study to measure the effect of post-operative physiotherapy intervention on different smoking groups. To increase the validity of the results and to limit possible bias, the physiotherapy team was blinded to the pre- and post-operative results and followed the exact treatment protocol. Also, the primary investigator was not included in patient treatment. Finally, the ICU consultant was blind to the X-rays after the patient was discharged from the ICU.

However, a limitation of this study is the exclusion of female participants, which introduces a potential selection bias. Despite this, the results did not significantly differ from other articles. Another limitation is that ICU LOS may be affected by available beds in the surgical step-down ward. Therefore, future studies are needed to overcome these limitations.

### Clinical implications

We must again mention that this is the first study to measure the effect of post-operative physiotherapy intervention on different smoking groups. Clinically, the results of this study may help understand the need to improve the therapeutic plan after CABG and establish a rehabilitation program and smoking cessation program before surgery. The results may also aid in decision-making regarding specific programs or the need to increase the number of sessions for certain patients. Additionally, a deeper understanding of the effects of smoking could assist in more accurately estimating care costs for such episodes.

## CONCLUSION

This quasi-experimental study measured the effect of post-operative physiotherapy intervention on different smoking groups. It was observed that smoking status significantly influenced functional capacity post-CABG, as evidenced by differences in the 6MWT results, particularly when comparing smokers to non-smokers. Additionally, current smokers were more likely to require prolonged mechanical ventilation and experienced higher rates of atelectasis compared to other groups. However, the length of stay in the ICU did not show a significant correlation with smoking status. Future studies are recommended to investigate the effect of different physiotherapy interventions on smoking groups by isolating individuals with independent risk factors that could affect the outcomes.

## References

[1] Tsao CW, Aday AW, Almarzooq ZI, Anderson CAM (2023). Heart Disease and Stroke Statistics-2023 Update: A Report From the American Heart Association. Circulation.

[2] Al-Nozha MM, Arafah MR, Al-Mazrou YY, Al-Maatouq MA (2004). Coronary artery disease in Saudi Arabia. Saudi Med J.

[3] Alhabib KF, Batais MA, Almigbal TH, Alshamiri MQ (2020). Demographic, behavioral, and cardiovascular disease risk factors in the Saudi population: results from the Prospective Urban Rural Epidemiology study (PURE-Saudi). BMC Public Health.

[4] Benjamin EJ, Blaha MJ, Chiuve SE, Cushman M (2017). Heart Disease and Stroke Statistics-2017 Update: A Report From the American Heart Association. Circulation.

[5] Schwinger RHG (2021). Pathophysiology of heart failure. Cardiovasc Diagn Ther.

[6] Stroo JF, van Steenbergen GJ, van Straten AH, Houterman S, Soliman-Hamad MA (2023). Long-term Outcome of Reexploration for Bleeding After Coronary Artery Bypass Grafting. J Cardiothorac Vasc Anesth.

[7] Kanejima Y, Shimogai T, Kitamura M, Ishihara K, Izawa KP (2020). Effect of Early Mobilization on Physical Function in Patients after Cardiac Surgery: A Systematic Review and Meta-Analysis. Int J Environ Res Public Health.

[8] Yüksel A, Kan II, Yolgösteren A, Velioğlu Y (2017). Are the Early Postoperative Outcomes of Coronary Artery Bypass Grafting Surgery in Elderly Women Worse Compared to Men's?. Braz J Cardiovasc Surg.

[9] Yuksel A, Yolgosteren A, Kan II, Cayir MC (2018). A comparison of early clinical outcomes of off-pump and on-pump coronary artery bypass grafting surgery in elderly patients. Acta Chir Belg.

[10] Landoni G, Augoustides JG, Guarracino F, Santini F (2011). Mortality reduction in cardiac anesthesia and intensive care: results of the first International Consensus Conference. HSR Proc Intensive Care Cardiovasc Anesth.

[11] Noyez L, Kievit PC, van Swieten HA, de Boer MJ (2012). Cardiac operative risk evaluation: The EuroSCORE II, does it make a real difference?. Neth Heart J.

[12] Echahidi N, Pibarot P, Després JP, Daigle JM (2007). Metabolic syndrome increases operative mortality in patients undergoing coronary artery bypass grafting surgery. J Am Coll Cardiol.

[13] Karimi A, Ahmadi H, Davoodi S, Movahedi N (2008). Factors affecting postoperative morbidity and mortality in isolated coronary artery bypass graft surgery. Surg Today.

[14] Sadeghi N, Sadeghi S, Mood ZA, Karimi A (2002). Determinants of operative mortality following primary coronary artery bypass surgery. Eur J Cardiothorac Surg.

[15] Jones RH, Hannan EL, Hammermeister KE, Delong ER (1996). Identification of preoperative variables needed for risk adjustment of short-term mortality after coronary artery bypass graft surgery. The Working Group Panel on the Cooperative CABG Database Project. J Am Coll Cardiol.

[16] Ståhle E, Bergström R, Holmberg L, Nyström SO, Hansson HE (1991). Risk factors for operative mortality and morbidity in patients undergoing coronary artery bypass surgery for stable angina pectoris. Eur Heart J.

[17] Wang J, Xiao F, Li Y, Xin Wq (2011). [Risk factors for operative mortality in 1,098 patients with coronary artery bypass grafting surgery: a single center report]. Beijing Da Xue Xue Bao Yi Xue Ban.

[18] Wasir H, Mehta Y, Pawar M, Choudhary A (2006). Predictors of operative mortality following primary coronary artery bypass surgery. Indian Heart J.

[19] Yousefzadeh A, Chung F, Wong DT, Warner DO, Wong J (2016). Smoking Cessation: The Role of the Anesthesiologist. Anesth Analg.

[20] Warner DO (2000). Preventing postoperative pulmonary complications: the role of the anesthesiologist. Anesthesiology.

[21] Thun MJ, Carter BD, Feskanich D, Freedman ND (2013). 50-year trends in smoking-related mortality in the United States. N Engl J Med.

[22] Heliövaara M, Karvonen MJ, Vilhunen R, Punsar S (1978). Smoking, carbon monoxide, and atherosclerotic diseases. Br Med J.

[23] Price JF, Mowbray PI, Lee AJ, Rumley A (1999). Relationship between smoking and cardiovascular risk factors in the development of peripheral arterial disease and coronary artery disease: Edinburgh Artery Study. Eur Heart J.

[24] Park SJ, Foreman MG, Demeo DL, Bhatt SP (2015). Menthol cigarette smoking in the COPDGene cohort: relationship with COPD, comorbidities and CT metrics. Respirology.

[25] Bassiony MM (2009). Smoking in Saudi Arabia. Saudi Med J.

[26] Moradi-Lakeh M, El Bcheraoui C, Tuffaha M, Daoud F (2015). Tobacco consumption in the Kingdom of Saudi Arabia, 2013: findings from a national survey. BMC Public Health.

[27] Mozaffarian D, Benjamin EJ, Go AS, Arnett DK (2016). Heart Disease and Stroke Statistics-2016 Update: A Report From the American Heart Association. Circulation.

[28] Wilson K, Gibson N, Willan A, Cook D (2000). Effect of smoking cessation on mortality after myocardial infarction: meta-analysis of cohort studies. Arch Intern Med.

[29] Morris RW, McCallum AK, Walker M, Whincup PH, Ebrahim S, Shaper AG (1996). Cigarette smoking in British men and selection for coronary artery bypass surgery. Heart.

[30] Odom NJ, Ashraf SS, Sharif MH, Akhtar K (1993). Access to heart surgery for smokers. Persuade smokers to give up before surgery. BMJ.

[31] Ashraf MN, Mortasawi A, Grayson AD, Oo AY (2004). Effect of smoking status on mortality and morbidity following coronary artery bypass surgery. Thorac Cardiovasc Surg.

[32] Al-Sarraf N, Thalib L, Hughes A, Tolan M (2008). Lack of correlation between smoking status and early postoperative outcome following valve surgery. Thorac Cardiovasc Surg.

[33] Utley JR, Leyland SA, Fogarty CM, Smith WP (1996). Smoking is not a predictor of mortality and morbidity following coronary artery bypass grafting. J Card Surg.

[34] Caram LM, Ferrari R, Bertani AL, Garcia T (2016). Smoking and Early COPD as Independent Predictors of Body Composition, Exercise Capacity, and Health Status. PLoS One.

[35] Weissman C (2004). Pulmonary complications after cardiac surgery. Semin Cardiothorac Vasc Anesth.

[36] Fischer MO, Brotons F, Briant AR, Suehiro K (2022). Postoperative Pulmonary Complications After Cardiac Surgery: The VENICE International Cohort Study. J Cardiothorac Vasc Anesth.

[37] Smetana GW, Lawrence VA, Cornell JE, American College of Physicians (2006). Preoperative pulmonary risk stratification for noncardiothoracic surgery: systematic review for the American College of Physicians. Ann Intern Med.

[38] Canet J, Gallart L, Gomar C, Paluzie G (2010). Prediction of postoperative pulmonary complications in a population-based surgical cohort. Anesthesiology.

[39] Shander A, Fleisher LA, Barie PS, Bigatello LM (2011). Clinical and economic burden of postoperative pulmonary complications: patient safety summit on definition, risk-reducing interventions, and preventive strategies. Crit Care Med.

[40] Agostini P, Naidu B, Cieslik H, Rathinam S (2011). Comparison of recognition tools for postoperative pulmonary complications following thoracotomy. Physiotherapy.

[41] Saxena A, Shan L, Reid C, Dinh DT (2013). Impact of smoking status on early and late outcomes after isolated coronary artery bypass graft surgery. J Cardiol.

[42] Patterson TL, Mausbach BT (2010). Measurement of functional capacity: a new approach to understanding functional differences and real-world behavioral adaptation in those with mental illness. Annu Rev Clin Psychol.

[43] Herdy AH, Marcchi PL, Vila A, Tavares C (2008). Pre-and postoperative cardiopulmonary rehabilitation in hospitalized patients undergoing coronary artery bypass surgery: a randomized controlled trial. Am J Phys Med Rehabil.

[44] Ramos Dos Santos PM, Aquaroni Ricci N, Aparecida Bordignon Suster É, de Moraes Paisani D, Dias Chiavegato L (2017). Effects of early mobilisation in patients after cardiac surgery: a systematic review. Physiotherapy.

[45] Mayr S, Erdfelder E, Buchner A, Faul F (2007). A Short Tutorial of G Power. Tutorials in Quantitative Methods for Psychology.

[46] Alam M, Siddiqui S, Lee VV, Elayda MA (2011). Isolated coronary artery bypass grafting in obese individuals: a propensity matched analysis of outcomes. Circ J.

[47] Holland AE, Spruit MA, Troosters T, Puhan MA (2014). An official European Respiratory Society/American Thoracic Society technical standard: field walking tests in chronic respiratory disease. Eur Respir J.

[48] Wilson RC, Jones PW (1989). A comparison of the visual analogue scale and modified Borg scale for the measurement of dyspnoea during exercise. Clin Sci (Lond).

[49] Olper L, Cervi P, De Santi F, Meloni C, Gatti R (2011). Validation of the treadmill Six-Minute Walk Test in people following cardiac surgery. Phys Ther.

[50] Abbott TEF, Fowler AJ, Pelosi P, Gama de Abreu M (2018). A systematic review and consensus definitions for standardised end-points in perioperative medicine: pulmonary complications. Br J Anaesth.

[51] Miskovic A, Lumb AB (2017). Postoperative pulmonary complications. Br J Anaesth.

[52] Chumillas S, Ponce JL, Delgado F, Viciano V, Mateu M (1998). Prevention of postoperative pulmonary complications through respiratory rehabilitation: a controlled clinical study. Arch Phys Med Rehabil.

[53] Hirschhorn AD, Richards D, Mungovan SF, Morris NR, Adams L (2008). Supervised moderate intensity exercise improves distance walked at hospital discharge following coronary artery bypass graft surgery--a randomised controlled trial. Heart Lung Circ.

[54] Garshick MS, Vaidean GD, Vani A, Underberg JA (2019). Cardiovascular Risk Factor Control and Lifestyle Factors in Young to Middle-Aged Adults with Newly Diagnosed Obstructive Coronary Artery Disease. Cardiology.

[55] Krittanawong C, Kumar A, Wang Z, Narasimhan B (2020). Coronary artery disease in the young in the US population-based cohort. Am J Cardiovasc Dis.

[56] Er Dedekargınoğlu B, Ulubay G, Küpeli E, Kırnap M (2016). Smoking Is Related to Postoperative Pulmonary Complications and Graft Outcomes in Renal Transplant Patients. Exp Clin Transplant.

[57] Ji Q, Zhao H, Mei Y, Shi Y (2015). Impact of smoking on early clinical outcomes in patients undergoing coronary artery bypass grafting surgery. J Cardiothorac Surg.

[58] Sathyaprasad SL, Thomas M, Philip FA, Krishna KJ (2020). Performance in 6-min walk test in prediction of post-operative pulmonary complication in major oncosurgeries: A prospective observational study. Indian J Anaesth.

[59] Santos BF, Souza HC, Miranda AP, Cipriano FG, Gastaldi AC (2016). Performance in the 6-minute walk test and postoperative pulmonary complications in pulmonary surgery: an observational study. Braz J Phys Ther.

[60] Guan Z, Lv Y, Liu J, Liu L (2016). Smoking Cessation Can Reduce the Incidence of Postoperative Hypoxemia After On-Pump Coronary Artery Bypass Grafting Surgery. J Cardiothorac Vasc Anesth.

[61] Atoui R, Ma F, Langlois Y, Morin JF (2008). Risk factors for prolonged stay in the intensive care unit and on the ward after cardiac surgery. J Card Surg.

[62] Richey M, Mann A, He J, Daon E (2018). Implementation of an Early Extubation Protocol in Cardiac Surgical Patients Decreased Ventilator Time But Not Intensive Care Unit or Hospital Length of Stay. J Cardiothorac Vasc Anesth.

[63] Zarrizi M, Paryad E, Khanghah AG, Leili EK, Faghani H (2021). Predictors of Length of Stay in Intensive Care Unit after Coronary Artery Bypass Grafting: Development a Risk Scoring System. Braz J Cardiovasc Surg.

[64] Dominici C, Salsano A, Nenna A, Spadaccio C (2020). A Nomogram for Predicting Long Length of Stay in The Intensive Care Unit in Patients Undergoing CABG: Results From the Multicenter E-CABG Registry. J Cardiothorac Vasc Anesth.

[65] Herdy AH, Marcchi PL, Vila A, Tavares C (2008). Pre-and postoperative cardiopulmonary rehabilitation in hospitalized patients undergoing coronary artery bypass surgery: a randomized controlled trial. Am J Phys Med Rehabil.

[66] Borges DL, Silva MG, Silva LN, Fortes JV (2016). Effects of Aerobic Exercise Applied Early After Coronary Artery Bypass Grafting on Pulmonary Function, Respiratory Muscle Strength, and Functional Capacity: A Randomized Controlled Trial. J Phys Act Health.

